# Prevalence of imposter syndrome and its association with depression, stress, and anxiety among nursing students: a multi-center cross-sectional study

**DOI:** 10.1186/s12912-024-02414-w

**Published:** 2024-11-27

**Authors:** Ayman Mohamed El-Ashry, Samah Mohamed Taha, Eman Sameh Abd Elhay, Heba Abdel-Hamid Hammad, Mahmoud Abdelwahab Khedr, Mona Metwally El-Sayed

**Affiliations:** 1 Psychiatric and Mental Health Nursing, Faculty of Nursing, Alexandria University, Alexandria, Egypt; 2 Psychiatric and Mental Health Nursing, Faculty of Nursing, Mansoura University, Mansoura, Egypt; 3 Psychiatric and Mental Health Nursing, Faculty of Nursing, Damanhour University, Damanhour, Egypt

**Keywords:** Imposter Syndrome, Depression, Anxiety, Stress, Nursing Students

## Abstract

**Objective:**

To examine the prevalence of imposter syndrome among nursing students and its association with depression, stress, and anxiety.

**Methods:**

A multi-center cross-sectional survey was conducted with 1572 nursing students randomly selected from three universities across Egypt. The study was conducted over 3 months, from the beginning of January to the end of March, during the academic year 2023/2024. Clance Imposter Phenomenon Scale and Depression Anxiety Stress Scale-21 were used for data collection.

**Results:**

A significant proportion of nursing students experience moderate to highly severe levels of depression, anxiety, and stress. Around a quarter of students had moderate depression, 22.6% had moderate anxiety, and 14.9% had severe anxiety. Additionally, 13.3% of participants experienced severe stress, with only 3.8% experiencing highly severe stress. The study also found that 46.3% of students experienced moderate imposter syndrome, with 33% having frequent imposter levels and 6.2% scoring intense imposter on the scale. Furthermore, imposter syndrome was positively correlated with depression, anxiety, and stress, and the total score of the Depression Anxiety Stress Scale (*r* = 0.639, *p* < 0.001). The stepwise linear regression analysis explained that 45.0% of the variance in students' depression, anxiety, and stress was due to imposter syndrome and other sociodemographic covariates.

**Conclusion:**

This study highlighted the relationship between imposter syndrome, depression, anxiety, and stress among nursing students. Specifically, female students in higher academic years, with lower grade point averages, living in urban areas, with lower family incomes, and not participating in hobbies are more prone to depression, anxiety, and stress.

**Nursing implications:**

These findings underscore the need for a holistic approach to managing the complex nature of mental health among nursing students. Incorporating education and resources on self-care and stress management techniques into nursing training programs is imperative for addressing the implications of imposter syndrome in nursing practice. Equipping nursing students with these essential skills can enhance their resilience, foster a healthier work environment, and ultimately improve patient care outcomes.

## Introduction

With its unique challenges and demanding nature, a nursing career requires a diverse skill set and a high level of competence. Extensive research has shown that nursing students, who face unique challenges such as managing disagreements with doctors, coping with heavy workloads, and interacting with patients and their families, are more susceptible to psychological issues like stress, anxiety, and depression compared to their peers in other fields [1–3]. In addition to these typical academic demands, nursing students encounter additional stressors during clinical practice [4].

Imposter Syndrome, also known as the Imposter Phenomenon (IP), is a prevalent experience among many nursing students. This psychological phenomenon, which leads them to believe that their achievements result from luck or deceit rather than their skills, causing them to fear being exposed to fraud, is a significant issue in nursing [6]. Research has consistently shown that nursing students experience higher levels of IP feelings than their professional counterparts [2, 7]. As a vulnerable group, nursing students are more likely to grapple with emotions related to IP, which can significantly impact their mental health and the quality of care they provide to their patients [8].

Imposter syndrome refers to the extreme feelings of dishonesty, fraudulence, and insecurity one experiences despite achieving something notable [2]. This feeling is prevalent, especially in settings where intelligence is crucial to success, such as academic environments. Even with objective proof of their success, people with imposter syndrome struggle to accept and internalize their accomplishments [9]. Imposter syndrome is common among nursing students who have just started their careers or switched from clinical practice to education [6]. This phenomenon can negatively affect mental health and career growth. Research shows that individuals experiencing imposter feelings may suffer from anxiety, sadness, and low self-esteem [10]. Nursing students who experience imposter syndrome may also exhibit higher levels of emotional distress and burnout, ultimately affecting their academic performance and professional development [5, 11].

Identifying the factors contributing to perceived fraudulence among nursing students is important. This understanding is crucial for addressing their mental health challenges during their educational journey [5]. Imposter syndrome could have profound implications for nursing education. Individuals who experience imposter syndrome are less likely to participate actively in class, whether by asking questions or sharing information. This could significantly impact their learning style and may necessitate educators to adapt the curriculum to cater to the needs of many learners with imposter syndrome [6]. Furthermore, nursing students experiencing IP may need help with clinical decision-making and confidence in their skills, potentially leading to suboptimal patient care and clinical outcomes [12]. The urgency of addressing imposter syndrome in nursing students cannot be overstated.

Previous research has shown that several factors are associated with the development and persistence of imposter syndrome. These include personal characteristics such as low self-esteem, critical self-assessment, and a tendency towards perfectionism. Additionally, nursing students may experience imposter syndrome due to external factors, including a competitive academic environment, high expectations from peers and teachers, and a lack of support networks [13, 14].

Studies have shown that the imposter phenomenon has yet to be thoroughly researched in nursing students [5, 11]. Additionally, the relationship between the imposter phenomenon and nursing students' depression, anxiety, and stress has not been fully explored. However, studies conducted in related fields, such as medical students and healthcare professionals, have highlighted the negative impact of imposter syndrome on mental health and general well-being. For example, Levant et al. (2020) found that medical students who experienced imposter feelings had higher levels of stress and anxiety. Similarly, Clark et al. [15] discovered a significant correlation between imposter syndrome and burnout in healthcare professionals [15].

### Significance of the study

This study addresses a critical gap in current research and has important nursing implications. The primary goal is to provide valuable insights that can inform targeted interventions and support strategies by uncovering the intricate relationship between Imposter Syndrome and depression, anxiety, and stress among nursing students. Through a deeper understanding of Imposter Syndrome and its effects, this study seeks to contribute to the development of holistic approaches to mental health management in nursing education, ensuring that future nurses are equipped with the necessary skills to navigate the challenges of their profession with confidence and competence. This study aimed to examine the prevalence of imposter syndrome among nursing students and its association with depression, stress, and anxiety.

### Research questions

- What is the levels and prevalence of imposter syndrome and depression, anxiety, and stress among undergraduate nursing students?
- What is the relationship between imposter syndrome and depression, anxiety, and stress among undergraduate nursing students, and what are the sociodemographic factors affecting this relationship?

## Methods

### Research design

We conducted a cross-sectional multiple centers survey, following the STROBE guidelines strictly.

### Setting

The study was conducted at three nursing colleges—Alexandria, Mansoura, and Damanhour—each operating under the jurisdiction of separate Egyptian authorities. Notably, the Ministry of Higher Education played a pivotal role in supervising these colleges' nursing programs, ensuring they adhered to Egyptian requirements for nursing education. Each college had nine scientific departments. The academic year, divided into three semesters: fall, spring, and summer, catered to both undergraduate and graduate students. The system of credit hours formed the foundation of the academic framework.

### Target Participants

#### Sample Size

The formula used for the calculation was = $\frac{\left(Z1- a/2\right).p(1-p)}{d2}$, where Z1 − α/2​ is 1.96 for 5% types 1 error, *p* < 0.05 is the expected proportion in the population based on previous studies such as Christensen et al. [5] and Klein, [16], and *d* is the absolute error or precision. Assuming a 10% unresponsive rate, the calculation indicated that a minimum sample size of 1,398 nursing students was required for the study [5, 16].

### Eligible criteria

To be eligible for participation, students had to meet certain criteria. Specifically, they needed to be enrolled as undergraduate students at the mentioned nursing colleges for the entire academic year 2023–2024 and express their voluntary willingness to participate in the research. However, the study excluded students who disclosed any pre-existing mental health conditions or declined to participate in the survey.

### Recruitment Process

The research was conducted over three months, from November 2023 to January 2024. A stratified random sampling method was used to select a representative sample of undergraduate nursing students. The Candidates' Affairs Department at each nursing college provided a comprehensive list of undergraduate students, which served as the sampling frame for the study. The students were divided into strata based on the total number of students in each college. The total number of enrolled students was 6731 for Alexandria University (A), 4842 for Damanhour University (B), and 7126 for Mansoura University (C), making a total of 18,699 students. Using the Random Generator software, 1638 participants were randomly selected across groups, ensuring a proportional representation from each university. However, 25 students (8 from A, 11 from B, and 6 from C) were excluded from the study as they self-reported having psychiatric disorders. Moreover, 27 students (10 from A, 9 from B, and 8 from C) refused to participate in the research, and 14 participants (4 from A, 7 from B, and 3 from C) initially agreed to participate but later withdrew, leaving a final sample of 1,572 students. This included 526 students from A, 307 from B, and 739 from C.

### Measurements

#### A Sociodemographic and academic datasheet

The survey includes questions about students' age, gender, marital status, region of residence, monthly family income, year of study, last Grade Point Average (GPA), and hobbies.

### Clance Imposter Phenomenon Scale (CIPS)

The Clance Impostor Phenomenon Scale (CIPS) is a widely used tool to measure the severity of imposter syndrome. It consists of 20 items, each rated on a 5-point Likert scale ranging from 1 (not at all true) to 5 (very true), with total scores ranging from 20 to 100. Scores less than 40 indicate low impostor characteristics, scores between 41 and 60 suggest moderate impostor experiences, scores between 61 and 80 indicate frequent impostor feelings, and scores over 80 denote intense impostor experiences [17]. The CIPS has demonstrated good content validity, meaning the items effectively cover the construct of imposter syndrome, and satisfactory construct validity, indicating that the scale measures what it intends to measure [18]. The internal consistency reliability of the CIPS is high, with Cronbach’s alpha values typically above 0.90, indicating that the items on the scale are highly correlated and measure the same underlying construct [18]. The CIPS does not have distinct subscales; it is designed to provide a single overall score reflecting the severity of imposter syndrome.

### Depression Anxiety Stress Scale-21 (DASS-21)

The Depression Anxiety Stress Scale-21 (DASS-21), translated into Arabic by Ali et al. in 2017, requires that the results be multiplied by 2 to align with the original DASS-42 scoring system [19]. This alignment is crucial as it ensures consistency in interpreting the severity of symptoms across its three subscales: depression, anxiety, and stress. The DASS-21, when adjusted, provides accurate and reliable measurements of mental health symptoms. Its depression Subscale assesses symptoms such as dysphoria, hopelessness, devaluation of life, self-deprecation, lack of interest/involvement, anhedonia, and inertia. The Anxiety Subscale evaluates symptoms related to autonomic arousal, skeletal muscle effects, situational anxiety, and subjective experience of anxious affect. The Stress Subscale measures symptoms associated with difficulty relaxing, nervous arousal, being easily upset/agitated, being irritable/over-reactive, and being impatient. After multiplication, each subscale score ranges from 0 to 42, with higher scores indicating greater severity. The DASS-21 has demonstrated good reliability and validity, with high internal consistency (Cronbach’s alpha values typically above 0.90) and discriminative solid, concurrent, and convergent validity [19].

### Procedure

#### Ethical considerations

The study was given ethical permission before starting by the Damanhour University College of Nursing's Research Ethics Committee (REC). The relevant organizations at each nursing college engaged in the study also gave additional ethical permits. The study received written support from the vice deans of the three universities. Each participant gave written informed permission and voluntarily consented to participate in the study. The research team implemented stringent protocols to ensure the confidentiality of the study participants. The study team was the only one with access to personal information collected, protecting the participants' privacy and anonymity. Every participant was aware of their freedom to leave the research at any time.

#### Validity and reliability

After obtaining consent, Confirmatory Factor Analysis (CFA) was used to confirm the accuracy of the Arabic translation of the CIPS. The Arabic version of the scale was translated, and CFA was used to verify its correctness. This produced goodness-of-fit indices; including a 0.951 Comparative Fit Index (CFI), a 0.980 Tucker-Lewis Index (TLI), and a 0.100 Root Mean Square Error of Approximation (RMSEA). According to the findings of this pilot study, 36 students (22.5%) and 43 students (26.8%) out of the total showed notable levels of stress, anxiety, and despair, as well as severe impostor syndrome symptoms. The internal consistency of the study instruments was assessed using Cronbach’s Alpha. The Cronbach’s alpha value for CIPS was 0.82, and for DASS-21, it was 0.84.

#### Pilot study

An initial investigation evaluated 120 undergraduate nursing students, but they were not included in the final group. The aim of the pilot study was to identify any necessary adjustments or modifications. However, no modifications were needed as the instruments were considered appropriate, comprehensible, and practical.

#### Data collection

After excluding the pilot study and before data collection, all data collectors underwent comprehensive training to ensure a standardized approach throughout the study. The training sessions covered the study's objectives, data collection instruments, and specific protocols to be followed in various scenarios. Additionally, practice sessions were conducted to allow data collectors to familiarize themselves with the tools and techniques, ensuring uniform application of the procedures. Interrater reliability was emphasized to ensure consistency and minimize variability in the data collection process. All data collectors were trained to use the same techniques and adhere strictly to the established protocols.

The trained researchers conducted structured interviews with each participant at their respective colleges. These interviews used approved instruments for data collection, ensuring a standardized approach across all participants. Each interview, lasting approximately 10–15 min, was held in a private classroom. This setting was chosen to maintain confidentiality and encourage candid responses, ensuring the participants' privacy and comfort were respected. The researchers were selected to ensure they had no authority over the students to avoid potential bias or influence.

Additionally, no incentives were provided to the participants, ensuring their voluntary involvement without external motivation. To maintain the accuracy and completeness of the collected data, the researchers thoroughly reviewed the responses immediately after each interview. This review process was crucial for identifying inconsistencies or incomplete answers, allowing immediate clarification, and ensuring high-quality data. The data collection period spanned three months, from the beginning of January to the end of March, during the academic year 2023/2024.

#### Data analysis and processing

The information gathered for the research was entered into IBM SPSS version 26 for examination [20]. The data was checked thoroughly to ensure accuracy and remove any possible errors in data input. The distribution of the quantitative variables was examined for normalcy using the Shapiro–Wilk and Kolmogorov–Smirnov tests. The two quantitative measures, DASS and CIPS, were assessed for internal consistency using Cronbach's alpha values. A confirmatory factor analysis was performed to check the accuracy of the CIPS translation into Arabic [21].

Descriptive statistics were employed in a thorough manner to present the imposter syndrome and depression, anxiety, and stress of the participants, including means, standard deviations, frequencies, and percentages. In this study, we meticulously conducted a stepwise linear regression analysis to explore the relationship between the imposter phenomenon and the total DASS-21 score, alongside other variables such as academic year, family income, gender, residence, and GPA. Before running the regression, we rigorously checked vital assumptions to ensure the validity of the results. The assumption of linearity was verified through scatterplots of the residuals, which indicated a linear relationship between the dependent variable (total DASS-21 score) and the independent variables (e.g., CIPS score, academic year). The Durbin-Watson statistic confirmed the independence of errors, which suggested no autocorrelation. Homoscedasticity was assessed through residual plots, demonstrating that the variance of residuals was constant across levels of the independent variables. The normality of residuals was examined using a Q-Q plot and confirmed by a non-significant Shapiro–Wilk test, indicating that the residuals were approximately normally distributed. Lastly, multicollinearity was evaluated using the variance inflation factor (VIF), with all values falling below the threshold of 10, indicating no significant multicollinearity. All statistical tests were executed at a significance level of 0.05 [22].

## Results

### Characteristics of nursing students

Table 1 shows that most of these students were between 20 and 22 (53.80%), while 40.50% were under 20. Regarding gender, there were more female students (66.70%) than male students (33.30%). Academic year, the largest group was in their first year (38.30%), followed by fourth-year students (23.10%). The students were almost evenly split between urban (50.8%) and rural (49.20%) residences. Most students had sufficient family income (61.20%), with some even able to save (29.60%). The vast majority of students were single (96.30%). Regarding their GPA, most students had a GPA between 3 and 4 (78.50%).

**Table 1:** Distribution of participants' demographic characteristics (*n* = 1572)

Variables	Total (n = 1572)	
	**No**	**%**
**Age**		
< 20	636	40.5%
20 – 22	845	53.8%
> 22	91	5.8%
**Gender**		
Males	524	33.3%
Females	1048	66.7%
**Academic Year**		
1st	601	38.3%
2nd	329	21.0%
3rd	278	17.7%
4th	362	23.1%
**Residence**		
Urban	799	50.8%
Rural	773	49.2%
**Family Income**		
Not Sufficient	145	9.2%
Sufficient	962	61.2%
Sufficient and saving	465	29.6%
**Marital Status**		
Single	1512	96.3%
Married	56	3.6%
Divorced	2	0.1%
**GPA (maximum 5)**		
1 – < 2	12	0.8%
2—< 3	254	16.2%
3—< 4	1234	78.5%
≥ 4	72	4.6%
**Hobbies**		
None	147	9.4%
Arts	391	24.9%
Computer Programmers	46	2.9%
Reading	134	8.5%
Handwork art	452	28.8%
Sports	402	25.6%

### Levels and Prevalence of imposter syndrome, depression, anxiety, and stress levels among nursing students

Table 2 shows that mild depression was observed in 16.20% of the students. Moderate depression was more prevalent, affecting 24.10% of the students. Severe and highly severe depressions were less common, affecting 9.20% and 6.80% of the students. Regarding anxiety level, it was found that a smaller proportion, 7.20%, exhibited mild anxiety. Moderate anxiety was observed in 22.60% of the participants. Severe anxiety was noted in 14.90% of the participants. The remaining 21.60% of the participants were found to have highly severe anxiety. Concerning stress levels, it was observed that mild stress was found in 14.60% of the students. Moderate stress was identified in 14.20% of the participants. Severe stress was experienced by 13.30% of the participants. The most minor proportion, 3.80%, was found to have highly severe stress.

**Table 2:** Distribution of Depression, Anxiety and Stress Scale—21 Items (DASS-21) (*n* = 1572)

DASS-21	Total sample (n = 1572)	
	**No**	**%**
**Depression**		
Normal	688	43.8%
Mild	254	16.2%
Moderate	379	24.1%
Severe	144	9.2%
Extremely Severe	107	6.8%
**Mean ± SD of Depression subscale**	**11.89 ± 9.12**	
**Anxiety**		
Normal	531	33.8%
Mild	113	7.2%
Moderate	355	22.6%
Severe	234	14.9%
Extremely Severe	339	21.6%
**Mean ± SD of Anxiety subscale**	**12.50 ± 8.98**	
**Stress**		
Normal	850	54.1%
Mild	229	14.6%
Moderate	224	14.2%
Severe	209	13.3%
Extremely Severe	60	3.8%
**Mean ± SD of Stress subscale**	**14.88 ± 9.56**	
**Total DASS-21**		
**Mean ± SD**	**39.27 ± 25.98**	

Table 3 provides the distribution of scores on the CIPS. It was found that 14.60% of nursing students scored low on the scale. Most students, 46.30%, had moderate imposter. Frequently, imposter level was observed in 33% of the nursing students. The smallest group, 6.10%, scored intense imposter on the scale.

**Table 3:** Distribution of Impostor syndrome scale (*n* = 1572)

Impostor syndrome scale	Total sample (n = 1572)	
	**No**	**%**
low	229	14.6%
Moderate	728	46.3%
Frequently	518	33.0%
Intense	97	6.2%
**Mean ± SD**	**56.65 ± 15.22**	

### The relationship between students’ socio-demographic data, imposter syndrome, depression, anxiety, and stress

Table 4 displays that the total score of the CIPS was found to be positively correlated with depression (*r *= 0.609*, p* < 0.001), anxiety (*r* = 0.599,* p* < 0.001), stress (*r* = 0.594, *p* < 0.001), and the total score of DASS-21 (*r* = 0.639, *p* < 0.001). The results indicated that higher scores on the CIPS were positively correlated with elevated levels of depression, anxiety, stress, and overall DASS-21 scores.

**Table 4:** Correlation coefficient between depression, anxiety, stress, and imposter syndrome among the participants (*n* = 1572)

Variables		Depression	Anxiety	Stress	DASS-21
**Depression**	r				
	p				
**Anxiety**	r	0.850*			
	p	< 0.001			
**Stress**	r	0.804*	0.820*		
	p	< 0.001	< 0.001		
**DASS-21**	r	0.940*	0.945*	0.933*	
	P	< 0.001	< 0.001	< 0.001	
**CIPS**	r	0.609*	0.599*	0.594*	0.639*
	P	< 0.001	< 0.001	< 0.001	< 0.001

DASS-21: Depression, Anxiety, and Stress Scale—21 Items

*CIPS* Clance Imposter Phenomenon Scale

*r*: Pearson coefficient, *: Statistically significant at p ≤ 0.05

Table 5 illustrates that students aged over 22 had the highest mean DASS-21 score (44.57 ± 23.88), with a statistically significant difference (*F* = 28.157, *p* < 0.001). Female students exhibited higher mean DASS-21 scores (42.68 ± 24.97) compared to male students (32.38 ± 26.64), and this difference was also significant (t = 7.542, *p* < 0.001). As students advanced through their academic years, the mean DASS-21 score increased from 32.48 ± 23.92 in the first year to 45.52 ± 23.98 in the fourth year, showing a statistically significant trend (*F* = 26.492, *p* < 0.001). Urban students had higher mean DASS-21 scores (44.10 ± 24.76) than rural students (34.21 ± 26.29), with a significant difference (t = 7.680, *p* < 0.001). Family income level significantly correlated with DASS-21 scores, where students with "insufficient" family income had the highest mean score (48.94 ± 33.10) (*F* = 13.077, *p* < 0.001). Regarding GPA, students with a GPA of 2–3 had the highest mean DASS-21 score (45.83 ± 26.78), and this difference was significant (*F* = 7.683, *p* < 0.001). hobbies also impacted DASS-21 scores significantly; students without hobbies had the highest mean score (46.61 ± 26.44), while those engaged in computer programming (27.35 ± 25.37) and sports (31.58 ± 24.87) had the lowest scores (*F* = 13.830, *p* < 0.001).

**Table 5:** Relationship between the participants' demographic characteristics and DASS_21 (*n* = 1572)

Students’ demographic characteristics	DASS-21
	**M (SD)**
**Age**	
< 20	33.40 ± 24.70
20 – 22	43.07 ± 26.34
> 22	44.57 ± 23.88
**F (p)**	28.157* (< 0.001)
**Gender**	
Males	32.38 ± 26.64
Females	42.68 ± 24.97
**t (p)**	7.542* (< 0.001)
**Academic Year**	
1st	32.48 ± 23.92
2nd	39.95 ± 26.16
3rd	45.01 ± 29.05
4th	45.52 ± 23.98
**F (p)**	26.492* (< 0.001)
**Residence**	
Urban	44.10 ± 24.76
Rural	34.21 ± 26.29
**t (p)**	7.680* (< 0.001)
**Family Income**	
Not enough	48.94 ± 33.10
Enough	39.16 ± 24.47
Enough and saving	36.40 ± 25.87
**F (p)**	13.077* (< 0.001)
**Marital status**	
Single	39.22 ± 26.21
Married	39.93 ± 20.08
Divorced	42.0 ± 0.0
**F (p)**	**0.031 (0.969)**
**GPA**	
1 – < 2	24.50 ± 16.27
2—< 3	45.83 ± 26.78
3—< 4	38.11 ± 25.64
≥ 4	37.83 ± 26.68
**F (p)**	7.683* (< 0.001)
**Recreational Activities**	
None	46.61 ± 26.44
Arts	41.33 ± 25.97
Computer Programmers	27.35 ± 25.37
Reading	42.27 ± 25.59
Handwork art	42.16 ± 25.22
Sports	31.58 ± 24.87
**F (p)**	13.830* (< 0.001)

DASS-21: Depression, Anxiety, and Stress Scale—21 Items

*F* One-way ANOVA test, *t* Student t-test, * Statistically significant at p ≤ 0.05

### The regression analysis of imposter syndrome and other covariates on depression, anxiety, and stress among students

Table 6 displays that in the first step, the CIPS was significantly associated with DASS-21, explaining 39.9% of the variance (adjusted *R*^2^ = 0.399). When the academic year was added in the second step, it was also significantly associated with DASS-21, increasing the explained variance to 42.9% (adjusted *R*^2^ = 0.429). In the third step, adding family income to the model revealed a significant negative association with DASS-21, slightly increasing the explained variance to 43.7% (adjusted *R*^2^ = 0.437). In the fourth step, gender inclusion in the model showed a significant association with DASS-21, raising the explained variance to 44.3% (adjusted *R*^2^ = 0.443). The fifth step added residence, which was significantly negatively associated with DASS-21, increasing the explained variance to 44.6% (adjusted *R*^2^ = 0.446). Finally, in the sixth step, GPA was added and found to be significantly associated with DASS-21, bringing the explained variance to 44.8% (adjusted *R*^2^ = 0.448). All factors included in the model were statistically significant at p ≤ 0.05.

**Table 6:** Stepwise Linear Regression Analysis Showing the Effect of imposter phenomenon and other covariates on Total DASS-21 (*n* = 1572)

	**B**	**Beta**	**t**	**p**	**95% CI**	
					**LL**	**UL**
**Step 1**						
CIPS	1.091	0.640	32.941*	< 0.001	1.026	1.156
***R***^**2**^** = 0.409, Adjusted** ***R***^**2**^** = 0.399,** *F* = 1085.11^*^, *p* < 0.001^*^						
**Step 2**						
CIPS	1.066	0.625	32.578*	< 0.001	1.001	1.130
Academic Year	3.174	0.145	7.585*	< 0.001	2.353	3.995
***R***^**2**^** = 0.430, Adjusted** ***R***^**2**^** = 0.429,** *F* = 590.888^*^, *p* < 0.001^*^						
**Step 3**						
CIPS	1.055	0.619	32.409*	< 0.001	0.992	1.119
Academic Year	3.347	0.153	8.019*	< 0.001	2.528	4.165
Family Income	-3.918	-0.089	-4.660*	< 0.001	-5.567	-2.269
***R***^**2**^** = 0.438, Adjusted** ***R***^**2**^** = 0.437,** *F* = 406.373^*^, *p* < 0.001^*^						
**Step 4**						
CIPS	1.038	0.609	31.834*	< 0.001	0.974	1.102
Academic Year	3.009	0.138	7.129*	< 0.001	2.181	3.837
Family Income	-4.205	-0.095	-5.014*	< 0.001	-5.850	-2.560
Gender	4.657	0.084	4.348*	< 0.001	2.556	6.757
***R***^**2**^** = 0.444, Adjusted** *R*^**2**^** = 0.443,** *F* = 312.991^*^, *p*** < 0.001**						
**Step 5**						
CIPS	1.029	0.603	31.492*	< 0.001	0.965	1.093
Academic Year	2.554	0.117	5.758*	< 0.001	1.684	3.424
Family Income	-4.197	-0.095	-5.020*	< 0.001	-5.837	-2.557
Gender	4.423	0.080	4.133*	< 0.001	2.324	6.522
Residence	-3.399	-0.065	-3.250*	< 0.001	-5.450	-1.348
***R***^**2**^** = 0.448, Adjusted** ***R***^**2**^** = 0.446,** *F* = 254.035^*^, ***p* < 0.001**						
**Step 6**						
CIP	1.030	0.604	31.576*	< 0.001	0.966	1.094
Academic Year	2.443	0.112	5.481*	< 0.001	1.569	3.318
Family Income	-4.235	-0.096	-5.071*	< 0.001	-5.873	-2.597
Gender	4.165	0.076	3.874*	< 0.001	2.056	6.273
Residence	-3.714	-0.071	-3.524*	< 0.001	-5.782	-1.647
GPA	2.488	0.043	2.237*	0.025	0.306	4.670
*R*^**2**^** = 0.450, Adjusted** ***R***^**2**^** = 0.448,** *F* = 213.072^*^, ***p* < 0.001**						

*CIP *Clance Imposter Phenomenon Scale, *F,p* f and p values for the model, *R*^2^ Coefficient of determination, *B* Unstandardized Coefficients, *Beta* Standardized Coefficients, *t* t-test of significance

*LL* Lower limit, *UL* Upper Limit, * Statistically significant at p ≤ 0.05

## Discussion

The psychological phenomenon known as "imposter syndrome," which causes people to question their successes and worry about being duped, is especially common among nursing students. Rather than attributing their achievements to their abilities and efforts, these students frequently battle feelings of inadequacy and self-doubt. The demands of nursing programs on students' academic performance, the hardships of living away from home, and financial constraints all contribute to this occurrence [23, 24]. The pervasive nature of imposter syndrome among nursing students not only impacts their mental health but also affects their academic performance and the quality of care they provide to patients. Understanding the factors contributing to imposter syndrome in this population is crucial for developing targeted interventions to support their mental well-being and professional development [24]. The current study examines the prevalence of imposter syndrome among nursing students and its association with depression, stress, and anxiety.

Our study sheds light on the prevalence of imposter syndrome among nursing students, as almost half of the participants reported moderate imposter syndrome. This can be attributed to the fact that nursing is a profession where even minor mistakes can have serious consequences, which puts added pressure on students to perform flawlessly. Anxiety over making mistakes or failing under pressure can contribute to the development of imposter syndrome among nursing students. These feelings can lead to overwhelm, uneasiness, insecurity, and uncertainty [11, 14]. Consistent with that, a study of 200 medical students found that 50.3%, 35.8%, and 6.7% experienced moderate, frequent, and intense imposter traits, respectively [25]. Another study found that students in medicine, dentistry, nursing, and pharmacy experienced the highest levels of imposter syndrome [26].

The findings revealed that more than one-third of the participants had normal levels of depression, while approximately two-thirds experienced moderate, severe, or extremely severe levels of depression. Regarding anxiety, approximately one-third had normal levels, and the other two-thirds of nursing students had moderate, severe, and highly severe anxiety. Additionally, a significant proportion of the participants experienced mild to moderate stress levels, while a smaller proportion reported severe to extremely severe stress. These mental health challenges could be attributed to the demanding nature of nursing, which requires students to make critical patient care decisions, a heavy workload, clinical responsibilities, concerns about the job market, balancing training and personal obligations, accumulating academic activities, evaluations, and more. These factors can make nursing students more susceptible to stress, anxiety, and depression [3, 4]. The incompetence could affect their ability to make informed decisions and prioritize tasks needed for their professional nursing practice. Students often express concerns about their ability to meet the demands of real-world healthcare facilities as they transition from classroom to clinical practice. With their high academic and clinical requirements, nursing programs can exacerbate feelings of inadequacy in students already overburdened by their expectations [27].

Recent research has shown that nursing students in Western countries are experiencing high levels of stress primarily due to academic demands. Basu et al. (2016) conducted a study on 129 nursing students in Kolkata using the DASS 21-point scale, which revealed that a significant percentage of students experienced depression, anxiety, and stress [28]. However, there is a gap in comparative research on stress, anxiety, and depression among Arab nursing students. Amr et al. [29] found that a considerable number of baccalaureate nursing students in Mansoura experienced stress, anxiety, and depression, with academic challenges being the primary cause of stress [29]. El-Ashry et al. [30] conducted a study in Egypt during COVID-19, which showed that perceived clinical stressors were evident among nursing students and were influenced by factors such as age, sex, study hours, and perceived worry from COVID-19 [30].

The present study uncovered a significant positive correlation between Imposter Syndrome and the psychological outcomes of depression, anxiety, and stress among nursing students. This relationship was substantiated through stepwise linear regression, which demonstrated that Imposter Syndrome significantly predicts these psychological outcomes. Additional contributors such as academic progression, socioeconomic status, gender, living arrangements, and academic performance were also highlighted. This correlation could be attributed to the cognitive and emotional processes involved in Imposter Syndrome. Individuals experiencing Imposter Syndrome often engage in negative self-evaluation, perfectionism, and fear of failure, which can exacerbate stress, anxiety, and depression [31, 32]. Our findings align with previous research demonstrating similar relationships. For instance, Saad et al. (2020) found a link between Imposter Syndrome and burnout among Saudi adults, illustrating the broader impact of Imposter Syndrome on mental health [33]. Moreover, Dong et al. [34] highlighted various stressors faced by nursing students, such as financial difficulties, lack of leisure activities, sleep issues, poor mental health, and working in general medicine, all contributing to elevated levels of depression, anxiety, and stress [34].

In the current study, academic progression contributed to more depressing anxiety and stress problems. This correlation can be attributed to the increasing academic demands and pressure to perform as students progress. That was consistent with Jacobs and Sasser [35], who reported a high prevalence of Imposter Syndrome and mental health problems among baccalaureate nursing students, further supporting our findings [35]. Contradictory, Holden et al. [36] observed that first-generation college students exhibit higher stress levels than their continuing-generation peers, indicating that certain demographic factors can intensify the effects of Imposter Syndrome [36].

In the current study, gender was another contributing factor for more depressing anxiety stress problems. Studies have shown that women are more likely to experience higher levels of Imposter Syndrome, which in turn can lead to increased rates of depression, anxiety, and stress [37]. This disparity may be due to societal pressures, gender stereotypes, and differing coping mechanisms between genders [38]. For instance, women often face higher expectations and harsher judgments in academic and professional settings, which can heighten feelings of inadequacy and impostorism [39]. In the Arab context, Altaweel et al. [40] found that moderate depression is prevalent among Saudi nursing students, particularly those under 23 who were dissatisfied with their nursing careers, highlighting the role of career satisfaction and age in mental health outcomes. Furthermore, they identified stress, GPA, and gender as significant predictors of depressive symptoms, mirroring the contributors identified in our study [40].

### Study limitations

This study has several limitations, even though it offers insightful information about how impostor syndrome affects nursing students' mental health. First, response bias might be present in the study because it depended on self-reported metrics. The fact that the study was restricted to nursing students from three Egyptian colleges may have limited the applicability of the findings to other demographics or cultural settings. Future research might broaden the sample to encompass nursing students from other nations and cultural backgrounds to better comprehend the occurrence. Future studies might examine how personality qualities, coping mechanisms, resilience, self-concealment, and social support play a part in this connection.

## Conclusion and recommendations

This study sheds light on the significant prevalence of depression, anxiety, stress, and imposter syndrome among nursing students. The study highlights the strong correlation between imposter syndrome, marked by feelings of intellectual fraudulence, and elevated levels of depression, anxiety, and stress. Demographic variables such as age, gender, academic year, GPA, residential area, monthly family income, and engagement in hobbies significantly influence mental health. Specifically, females, students in higher academic years, those with a lower GPA, residents of urban areas, and those with lower family incomes. These findings emphasize the multifaceted nature of mental health among nursing students, influenced by imposter syndrome and other factors, underscoring the need for a holistic approach to their management and elucidating the multifaceted nature of mental health challenges in nursing education.

### Nursing implications

The findings of this study have significant implications for nursing education and practice. Nurse educators should be aware of the prevalence of imposter syndrome among nursing students and its association with adverse mental health outcomes. Proactive steps should be taken to incorporate imposter syndrome screening and early intervention strategies into nursing curricula. This may involve fostering open dialogues about imposter feelings, providing counseling services, and equipping students with coping mechanisms to manage stress, anxiety, and depression [26]. Additionally, nurse educators can implement mentorship programs and peer support networks to help students navigate the challenges of nursing education and clinical practice, thereby mitigating the development of imposter syndrome [35].

Furthermore, healthcare organizations should prioritize the mental well-being of nursing students during their clinical placements. Providing accessible mental health resources, such as counseling services and stress management workshops, can help students develop resilience and overcome challenges [26]. Adopting a supportive and empathetic approach toward nursing students can create an environment that fosters their professional growth and minimizes the negative impact of imposter syndrome [25]. By addressing the mental health needs of nursing students, healthcare providers can contribute to developing competent and confident nurses who are better equipped to provide high-quality patient care [41–43].

## Data Availability

The datasets generated and/or analyzed during the current study are not publicly available due to restrictions imposed by the institutional review board to protect participant confidentiality, but are available from the corresponding author on reasonable request.

## References

[1] Al Maqbali M, Al Sinani M, Al-Lenjawi B. Prevalence of stress, depression, anxiety, and sleep disturbance among nurses during the COVID-19 pandemic: A systematic review and meta-analysis. J Psychosom Res. 2021;141: 110343. 10.1016/j.jpsychores.2020.110343. 33360329 10.1016/j.jpsychores.2020.110343 PMC7831768

[2] Bernard DL, Lige QM, Willis HA, Sosoo EE, Neblett EW. Impostor phenomenon and mental health: The influence of racial discrimination and gender. J Couns Psychol. 2017;64(2):155. https://psycnet.apa.org/10.1037/cou0000197 . 28182493 10.1037/cou0000197

[3] Bartlett ML, Taylor H, Nelson JD. Comparison of mental health characteristics and stress between baccalaureate and non-nursing students. J Nurs Educ. 2016;55(2):87–90. 10.3928/01484834-20160114-05. 26814818 10.3928/01484834-20160114-05

[4] Wang AH, Lee CT, Espin S. Undergraduate nursing students’ experiences of anxiety-producing situations in clinical practicums: A descriptive survey study. Nurse Educ Today. 2019;76:103–8. 10.1016/j.nedt.2019.01.016. 30776531 10.1016/j.nedt.2019.01.016

[5] Christensen M, Aubeeluck A, Fergusson D, Craft J, Knight J, Wirihana L, Stupple E. Do student nurses experience the Imposter Phenomenon? An International Comparison of Final Year Undergraduate Nursing Students readiness for registration. J Adv Nurs. 2016;72(11):2784–93. 10.1111/jan.13034. 27240171 10.1111/jan.13034

[6] John S. Imposter syndrome: Why some of us doubt our competence. Nurs Times. 2019;115(2):23. https://cronfa.swan.ac.uk/Record/cronfa46068.

[7] Brauer K, Proyer RT. Are impostors playful? Testing the association of adult playfulness with the impostor phenomenon. Pers Individ Dif. 2017;116:57–62. 10.1016/j.paid.2017.04.029.

[8] Gómez-Morales A. Impostor phenomenon: A concept analysis. Nurs Sci Q. 2021;34(3):309–15. 10.1177/08943184211010462. 34212809 10.1177/08943184211010462

[9] Parkman A. The imposter phenomenon in higher education: Incidence and impact. J High Educ Theory Pract. 2016;16(1).

[10] Sawant NS, Kamath Y, Bajaj U, Ajmera K, Lalwani D. A study on impostor phenomenon, personality, and self-esteem of medical undergraduates and interns. Ind Psychiatry J. 2023;32(1):136. 10.4103/2Fipj.ipj_59_22. 37274568 10.4103/ipj.ipj_59_22 PMC10236681

[11] Peng Y, Xiao S-W, Tu H, Xiong X-Y, Ma Z-J, Xu W-J, Cheng T. The impostor phenomenon among nursing students and nurses: a scoping review. Front Psychol. 2022;13: 809031. 10.3389/fpsyg.2022.809031. 35356345 10.3389/fpsyg.2022.809031 PMC8959846

[12] Villwock JA, Sobin LB, Koester LA, Harris TM. Impostor syndrome and burnout among American medical students: a pilot study. Int J Med Educ. 2016;7:364. 10.5116/2Fijme.5801.eac4. 27802178 10.5116/ijme.5801.eac4 PMC5116369

[13] Sheveleva MS, Permyakova TM, Kornienko DS. Perfectionism, the Impostor Phenomenon, Self-Esteem, and Personality Traits among Russian College Students. Psychol Russia. 2023;16(3):132. 10.11621/pir.2023.0310. 10.11621/pir.2023.0310 PMC10659233 38024563

[14] Shinawatra P, Kasirawat C, Khunanon P, Boonchan S, Sangla S, Maneeton B, et al. Exploring Factors Affecting Impostor Syndrome among Undergraduate Clinical Medical Students at Chiang Mai University, Thailand: A Cross-Sectional Study. Behav Sci. 2023;13(12):976. 10.3390/bs13120976. 38131833 10.3390/bs13120976 PMC10740738

[15] Clark P, Holden C, Russell M, Downs H. The impostor phenomenon in mental health professionals: relationships among compassion fatigue, burnout, and compassion satisfaction. Contemp Fam Ther. 2022;44(2):185–97. 10.1007/s10591-021-09580-y. 33948046 10.1007/s10591-021-09580-y PMC8085648

[16] Klein GJM. The relationships among anxiety, self-concept, the impostor phenomenon, and generic senior baccalaureate nursing students’ perceptions of clinical competency. Widener University School of Nursing; 2000.

[17] Clance PR. Clance impostor phenomenon scale. Personality Individ Differ. 1985. 10.1037/t11274-000.

[18] Chrisman SM, Pieper WA, Clance PR, Holland CL, Glickauf-Hughes C. Validation of the Clance imposter phenomenon scale. J Pers Assess. 1995;65(3):456–67. 10.1207/s15327752jpa6503_6. 16367709 10.1207/s15327752jpa6503_6

[19] Ali AM, Ahmed A, Sharaf A, Kawakami N, Abdeldayem SM, Green J. The Arabic Version of The Depression Anxiety Stress Scale-21: Cumulative scaling and discriminant-validation testing. Asian J Psychiatry. 2017;30:56–8. 10.1016/j.ajp.2017.07.018. 10.1016/j.ajp.2017.07.018 28755630

[20] IBM Corp. IBM SPSS Statistics for Windows, Version 26.0. Armonk, NY: IBM Corp; 2019.

[21] Brown TA. Confirmatory Factor Analysis for Applied Research. New York: Guilford Publications; 2015.

[22] Cohen J. Statistical Power Analysis for the Behavioral Sciences. 2nd ed. Hillsdale, NJ: Lawrence Erlbaum Associates; 1988.

[23] Cilar L, Barr O, Štiglic G, Pajnkihar M. Mental well-being among nursing students in Slovenia and Northern Ireland: A survey. Nurse Educ Pract. 2019;39:130–5. 10.1016/j.nepr.2019.07.012. 31476545 10.1016/j.nepr.2019.07.012

[24] Zhou L, Chankoson T, Wu Y, Cai E. Thriving psychological well-being in undergraduate nursing student: a grounded theory study with the life grid approach. BMC Nurs. 2023;22(1):240. 10.1186/s12912-023-01338-1. 37454074 10.1186/s12912-023-01338-1 PMC10349505

[25] Elnaggar M, Alanazi T, Alsayer NA, Alrawili M, Alanazi R, Alghamdi R, et al. Prevalence and Predictor of Impostor Phenomenon Among Medical Students at Jouf University, Saudi Arabia. Cureus. 2023;15(11). 10.7759/cureus.48866 10.7759/cureus.48866 PMC10724504 38106704

[26] Henning K, Ey S, Shaw D. Perfectionism, the imposter phenomenon, and psychological adjustments in medical, dental, nursing, and pharmacy students. Med Educ. 1998;32(5):456–64. 10.1046/j.1365-2923.1998.00234.x. 10211285 10.1046/j.1365-2923.1998.00234.x

[27] Aller L, Almarwani AM. Self-doubt in nursing students: An evolutionary concept analysis. Adv Nurs Sci. 2022. 10.1097/ans.0000000000000475. 10.1097/ANS.0000000000000475 36598385

[28] Basu M, Sinha D, Ahamed A, Chatterjee S, Misra RN. Depression, Anxiety, Stress among nursing students of Kolkata: a cross-sectional study. J Prev Med Holist Health. 2016;2(2):54–60. 10.18231/2454-6712.2016.0007.

[29] Amr A, El-Gilany AH, El-Moafee H, Salama L, Jimenez C. Stress among Mansoura (Egypt) baccalaureate nursing students. Pan Afr Med J. 2011;8:26. 10.4314/pamj.v8i1.71083. 22121435 10.4314/pamj.v8i1.71083 PMC3201591

[30] El-Ashry AM, Harby SS, Ali AAG. Clinical stressors as perceived by first-year nursing students of their experience at Alexandria main university hospital during the COVID-19 pandemic. Arch Psychiatr Nurs. 2022;41:214–20. 10.1016/j.apnu.2022.08.007. 36428052 10.1016/j.apnu.2022.08.007 PMC9433067

[31] Clance PR. The imposter phenomenon in high achieving women: Dynamics and therapeutic intervention. Psychother. 1978;15(3):241. https://psycnet.apa.org/10.1037/h0086006.

[32] Thompson T, Foreman P, Martin F. Impostor fears and perfectionistic concern over mistakes. Personality Individ Differ. 2000;29(4):629–47. 10.1016/S0191-8869(99)00218-4.

[33] Saad A, Dar UF, Musab A, Ahmed A, Nouf A. Burnout and imposter syndrome among Saudi young adults. Saudi Med J. 2020;41(2):189–94. 10.15537/smj.2020.2.24841. 32020154 10.15537/smj.2020.2.24841 PMC7841628

[34] Dong X, Yang K, Zhang R, Lv Y. The mental health and grade point average among college students from lower socioeconomic status based on healthcare data analysis. J Healthc Eng. 2021. 10.1155/2021/2378202. 34900179 10.1155/2021/2378202 PMC8654553

[35] Jacobs MD, Sasser JT. Impostor phenomenon in undergraduate nursing students: a pilot study of prevalence and patterns. J Nurs Educ. 2021;60(6):329–32. 10.3928/01484834-20210520-05. 34077317 10.3928/01484834-20210520-05

[36] Holden CL, Wright LE, Herring AM, Sims PL. Imposter Syndrome Among First- and Continuing-Generation College Students: The Roles of Perfectionism and Stress. J Coll Stud Retent. 2024;25(4):726–40. 10.1177/15210251211019379.

[37] Cokley K, McClain S, Enciso A, Martinez M. An examination of the impact of minority status stress and impostor feelings on the mental health of diverse ethnic minority college students. J Multicult Couns Dev. 2013;41(2):82–95. 10.1002/j.2161-1912.2013.00029.x.

[38] Young V. The secret thoughts of successful women: And men: Why capable people suffer from impostor syndrome and how to thrive in spite of it. Crown Currency; 2011;25.

[39] Hutchins HM. Outing the imposter: A study exploring imposter phenomenon among higher education faculty. New Horizons in Adult Education and Human Resource Development. 2015;27(2):3–12. 10.1002/nha3.20098.

[40] Altaweel F, Kamel N, Alqahtani F. Self-Esteem and Determinants of Depression among Undergraduate Nursing Students in Dammam, Saudi Arabia. Med Arch. 2023;77(1):44. 10.5455/medarh.2023.77.44-48. 36919131 10.5455/medarh.2023.77.44-48 PMC10008262

[41] Pervez A, Brady LL, Mullane K, Lo KD, Bennett AA, Nelson TA. An empirical investigation of mental illness, impostor syndrome, and social support in management doctoral programs. J Manag Educ. 2021;45(1):126–58. 10.1177/1052562920953195.

[42] El-Ashry AM, El-Sayed MM, Elhay ES, Taha SM, Atta MH, Hammad HA, Khedr MA. Hooked on technology: examining the co-occurrence of nomophobia and impulsive sensation seeking among nursing students. BMC Nurs. 2024;23(1):18. 10.1186/s12912-023-01683-1. 38166837 10.1186/s12912-023-01683-1 PMC10763039

[43] AlOtaibi NG, Alshowkan A, Kamel N, El-Ashry AM, AlSaleh NS, Abd Elhay ES. Assessing perceptions about critical thinking, motivation learning strategies in online psychiatric and mental health nursing education among Egyptian and Saudi undergraduate nursing students. BMC Nurs. 2023;22(1):112. 10.1186/s12912-023-01264-2. 37038179 10.1186/s12912-023-01264-2 PMC10084656

